# Potential prognostic and immunotherapeutic value of calponin 1: A pan-cancer analysis

**DOI:** 10.3389/fphar.2023.1184250

**Published:** 2023-04-21

**Authors:** Hengli Zhou, Junyu Ke, Changhua Liu, Menglu Zhu, Bijuan Xiao, Qi Wang, Rui Hou, Yueer Zheng, Yongqiang Wu, Xingting Zhou, Xinlin Chen, Huafeng Pan

**Affiliations:** ^1^ Science and Technology Innovation Center, Guangzhou University of Chinese Medicine, Guangzhou, China; ^2^ School of Basic Medical Science, Guangzhou University of Chinese Medicine, Guangzhou, China; ^3^ Gaozhou Hospital of Traditional Chinese Medicine, Gaozhou, China; ^4^ Namyue Natural Medicine Co., Ltd., Macau, Macau SAR, China; ^5^ Yu Fai Co., Ltd., Macau, Macau SAR, China; ^6^ Namyue Group Co., Ltd., Macau, Macau SAR, China; ^7^ Jiangsu Collaborative Innovation Center of Traditional Chinese Medicine in Prevention and Treatment of Tumor, Nanjing University of Chinese Medicine, Nanjing, China

**Keywords:** calponin 1, pan-cancer, immunotherapy, prognosis, angiogenesis, gastric cancer

## Abstract

**Background:** Emerging evidence has suggested a pro-oncogenic role of calponin 1 (CNN1) in the initiation of a variety of cancers. Despite this, CNN1 remains unknown in terms of its effects and mechanisms on angiogenesis, prognosis, and immunology in cancer.

**Materials and Methods:** The expression of CNN1 was extracted and analyzed using the TIMER, UALCAN, and GEPIA databases. Meanwhile, we analyzed the diagnostic value of CNN1 by using PrognoScan and Kaplan–Meier plots. To elucidate the value of CNN1 in immunotherapy, we used the TIMER 2.0 database, TISIDB database, and Sangerbox database. Gene set enrichment analysis (GSEA) was used to analyze the expression pattern and bio-progression of CNN1 and the vascular endothelium growth factor (VEGF) in cancer. The expressions of CNN1 and VEGF in gastric cancer were confirmed using immunohistochemistry. We used Cox regression analysis to investigate the association between pathological characteristics, clinical prognosis, and CNN1 and VEGF expressions in patients with gastric cancer.

**Results:** CNN1 expression was higher in normal tissues than it was in tumor tissues of most types of cancers. However, the expression level rebounds during the development of tumors. High levels of CNN1 indicate a poor prognosis for 11 tumors, which include stomach adenocarcinoma (STAD). There is a relationship between CNN1 and tumor-infiltrating lymphocytes (TILs), and the marker genes NRP1 and TNFRSF14 of TILs are significantly related to CNN1 expression in gastric cancers. The GSEA results confirmed the lower expression of CNN1 in tumors when compared to normal tissues. However, CNN1 again showed an increasing trend during tumor development. In addition, the results also suggest that CNN1 is involved in angiogenesis. The immunohistochemistry results validated the GSEA result (take gastric cancer as an example). Cox analysis suggested that high CNN1 expression and high VEGF expression are closely associated with poor clinical prognosis.

**Conclusion:** Our study has shown that CNN1 expression is aberrantly elevated in various cancers and positively correlates with angiogenesis and the immune checkpoint, contributing to cancer progression and poor prognosis. These results suggest that CNN1 could serve as a promising candidate for pan-cancer immunotherapy.

## Graphical Abstract

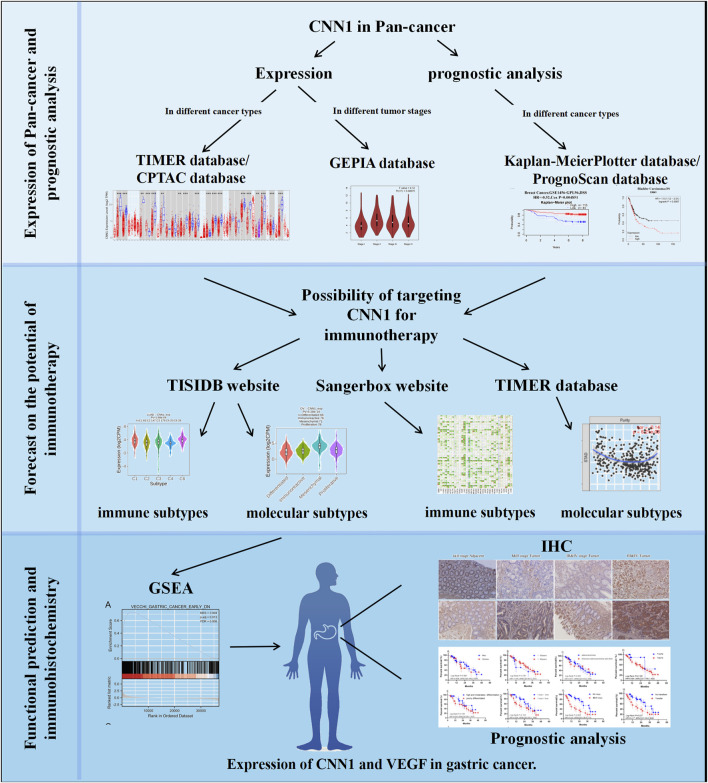

## 1 Introduction

The overall uptrend in the incidence and mortality of cancers remains a serious challenge globally that threatens human health and declines life quality. Over the decades, global joint projects, such as the Human Genome Project, Human Pathology Atlas, and Human Cell Atlas, have aimed to construct a series of online databases to uncover the pathogenesis of diseases and promote targeted drug developments and precision interventions. These progressions would be further promoted by the emergence of single-cell sequencing, spatial transcriptomics sequencing, and CAR-T immunotherapy. Recently, pan-cancer analysis has served as a novel hot spot to integrate multiple online databases to screen and investigate the key points and common molecular events linked to the occurrence and advancement of cancer (Cocco et al., 2018; Solomon et al., 2019). Therefore, the potential value of pan-cancer analyses is enormous. Gastric cancer (GC) is well-known as one of the most lethal cancers. Global Cancer Statistics 2020 has indicated that GC is the fifth most commonly diagnosed cancer (5.6%) and fourth leading cause of cancer-related mortality (7.7%) (Sung et al., 2021). The search for biomarkers that can be used for early diagnosis and treatment of cancer is necessary. Tumor immunotherapy, which is different from conventional chemotherapy and radiotherapy, focuses on regulating the immunogenicity of cancer or activating T cells to identify specific immune checkpoints of tumor cells and eliminate cancer cells. This is a method with great potential for high efficacy in cancer treatment (Yang, 2015).

CNN1 is a filament-associated protein located in the cytoskeleton, which is implicated in the maintenance of blood vessel integrity and regulation of smooth muscle contraction (Miano and Olson, 1996; Samaha et al., 1996). CNN1 was found to trigger the growth of “leaky” tumor vessels. CNN1 is also required for the maturation of tumor vessels, and its loss in host mural cells would inhibit this process (Yamamura et al., 2007). Moreover, CNN1 displays tumor-suppressive properties in human uterine leiomyosarcoma (Horiuchi et al., 1999). CNN1 deficiency causes structural fragility of blood vessels and the peritoneum and promotes hematogenous metastasis and peritoneal dissemination of malignant tumor cells (Taniguchi et al., 2001). Some studies have shown that CNN1 has an inhibitory effect on the malignant biological behavior of lung squamous cell carcinomas, such as invasion and migration, and also affects their epithelial–mesenchymal transition (Li et al., 2017). These findings have suggested that CNN1 would act as a double-edged sword in a complex context, which might depend on its expression level and tissue specificity.

In this study, we conducted a comprehensive pan-cancer analysis to summarize the prognostic significance of CNN1 in various types of cancer. Additionally, we explored the impact of CNN1 on mediating angiogenesis, immune checkpoint modulation, and prognostic value to assess its potential applications in targeted cancer intervention.

## 2 Materials and methods

### 2.1 CNN1 expression in pan-cancerous tissues and tumor staging

With the TCGA tumor data collected from the TIMER database (Li et al., 2017), a free web server built on R’s Shiny web framework (version 3.6.1), we used the exploration module of the website to investigate the expression of CNN1 in the TCGA pan-cancer cohort and presented it through the visualization function of the website.

The UALCAN database (Chandrashekar et al., 2022), a website that allows online exploration, analysis, and visualization of tumor data, contains the comprehensive tumor database TCGA and the tumor proteome database CPTAC. We used the website to analyze the differences in CNN1 expression across tumor groups in the CPTAC data set. We selected the CPTAC data set, entered “CNN1”, and analyzed the expression of CNN1 in breast cancer (BRCA), ovarian cancer (OV), colorectal adenocarcinoma (COAD), kidney renal clear cell carcinoma (KIRC), uterine corpus endometrial carcinoma (UCEC), lung adenocarcinoma (LUAD), head and neck squamous carcinoma (HNSC), pancreatic ductal adenocarcinoma (PAAD), and hepatocellular carcinoma (HCC).

We analyzed the relationship between CNN1 expression and tumor pathological stages (stages I–IV) based on the TCGA and GTEx data through the online GEPIA database (Tang et al., 2017). This was done by selecting the website and entering “CNN1” and selecting the StagePlot module, which transforms the expression data using the log2 (TPM + 1) log scale, and the results were visualized as violin plots.

### 2.2 Prognostic analysis of CNN1 based on different databases

To identify the effects of CNN1 expression on prognosis for various tumor types, we performed a prognostic analysis in the PrognoScan and Kaplan–Meier plotter databases (Mizuno et al., 2009; Nagy et al., 2018). By searching the databases for “CNN1”, we obtained the prognostic relationship between CNN1 expression levels and various tumor types, which included three different types of survival: overall survival (OS), disease-free survival (DFS), and disease-specific survival (DSS). Furthermore, based on the online survival analysis function provided by the Kaplan–Meier plotter database, we evaluated how CNN1 expression affects the prognosis of the different tumors. We entered “CNN1” in the abovementioned website, selected OS as a survival index, analyzed 21 types of tumors such as bladder carcinoma, and selected results with *p* < 0.05 for uniform presentation.

### 2.3 Relationship between CNN1 and immune cell infiltration and immune loci

The TISIDB platform explores tumor–immune interactions with data sets primarily derived from TCGA (Ru et al., 2019). Through the TISIDB website, we performed correlation analysis of the immune or molecular subtypes of CNN1 in different tumors. Meanwhile, we analyzed the relationship between CNN1 and immune loci in the Sangerbox database, which included a total of 47 common immune checkpoint genes, and presented the analysis results as heat maps through the website visualization function (Hu et al., 2021). TIMER 2.0 was used to analyze immune cell infiltration in tumor tissues to determine the relationship between CNN1 and immune cell infiltration (Li et al., 2020).

### 2.4 Functional enrichment analysis

The TCGA database was used to extract transcriptional expression profiles of 407 GC samples, and the C2 gene collection from the Molecular Signatures Database v 5.2 was used for the main analysis. Based on a gene set enrichment analysis (GSEA), we determined the function of the gene sets. When *p* < 0.05 and the false discovery rate (FDR) <0.25, the gene set was considered significantly enriched.

### 2.5 HE staining and immunohistochemical study of CNN1 in tumor tissues and paracancerous tissues

To make these findings more convincing, we included a total of 50 clinical GC samples for HE staining and immunohistochemical studies for CNN1 and VEGF expressions in clinical GC tissues. The human GC tumor specimens were obtained from the Department of Surgery II, Gaozhou Hospital of Traditional Chinese Medicine, Guangdong Province, and the Declaration of Helsinki was followed in all experimental contents of this study involving human tissues. This study was also approved by the Ethics Review Committee of Gaocheng City Hospital of Traditional Chinese Medicine (No. Y(2021)18). Before performing immunohistochemical studies, we embedded the tumor tissues in paraffin and performed HE staining. After rehydrating the tissue sections with xylene and alcohol, hematoxylin and eosin were used to label the nucleus and cytoplasm, and the pathological features of the tumor and paracancerous tissues were observed under the microscope (Lonardi et al., 2021). In the immunohistochemical study, the primary antibodies were anti-calponin 1 (Ab46794; Abcam) and anti-VEGF (Ab1316; Abcam), and each section was photographed at ×200 magnification with three fields of view selected to control the photographic conditions: shooting magnification, shooting brightness, white balance, and exposure time. Statistical analysis was performed using the ImageJ software to quantify the average optical density (AOD) value, i.e., Integrated Optical Density sum/area sum. Finally, the average optical density of the three fields of view in each slice was used as the optical density value of this sample.

### 2.6 Statistical and prognostic analyses of clinical information

The Fisher’s exact test and Wilcoxon rank-sum test were used for categorical and continuous type variables, respectively, to assess the relationship between the expression levels of CNN1, VEGF, and each clinical feature. The univariate Cox risk ratio regression was used to assess the discrete hazard ratio (HR) of CNN1 expression and other pathological features, and independent variables were sought. A *p* < 0.05 was considered to be a significant difference.

## 3 Results

### 3.1 Expression of CNN1 in pan-cancerous tissues

**FIGURE 1:**
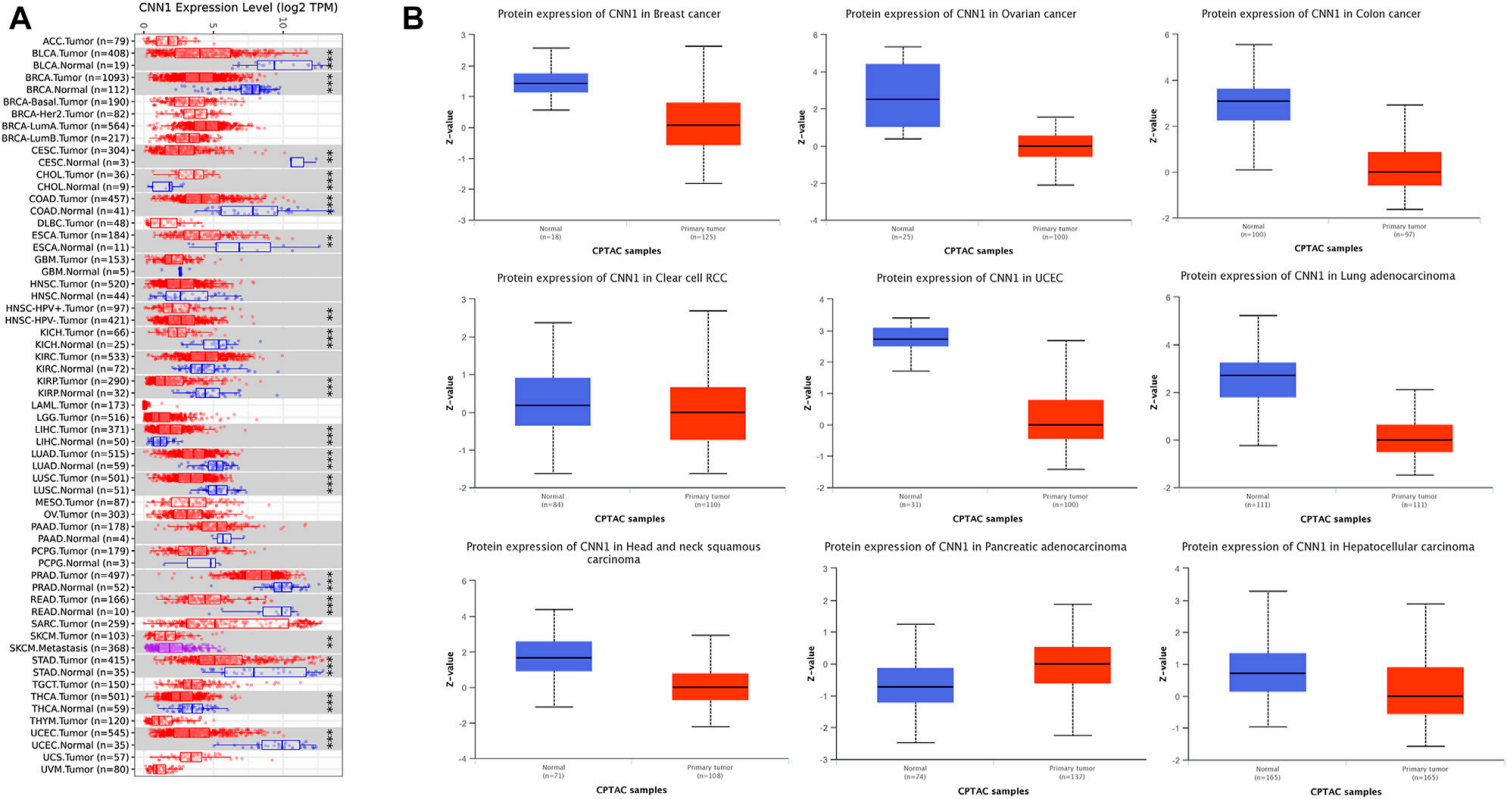
Analysis of CNN1 expression levels in human pan-cancer samples. **(A)** Expression levels of CNN1 in different cancer types in the TCGA database analyzed using the TIMER database (*p < 0.05, **p < 0.01, and ***p < 0.001). **(B)** Differences in CNN1 expression between tumor and paracancerous tissues in the CPTAC database.

**FIGURE 2:**
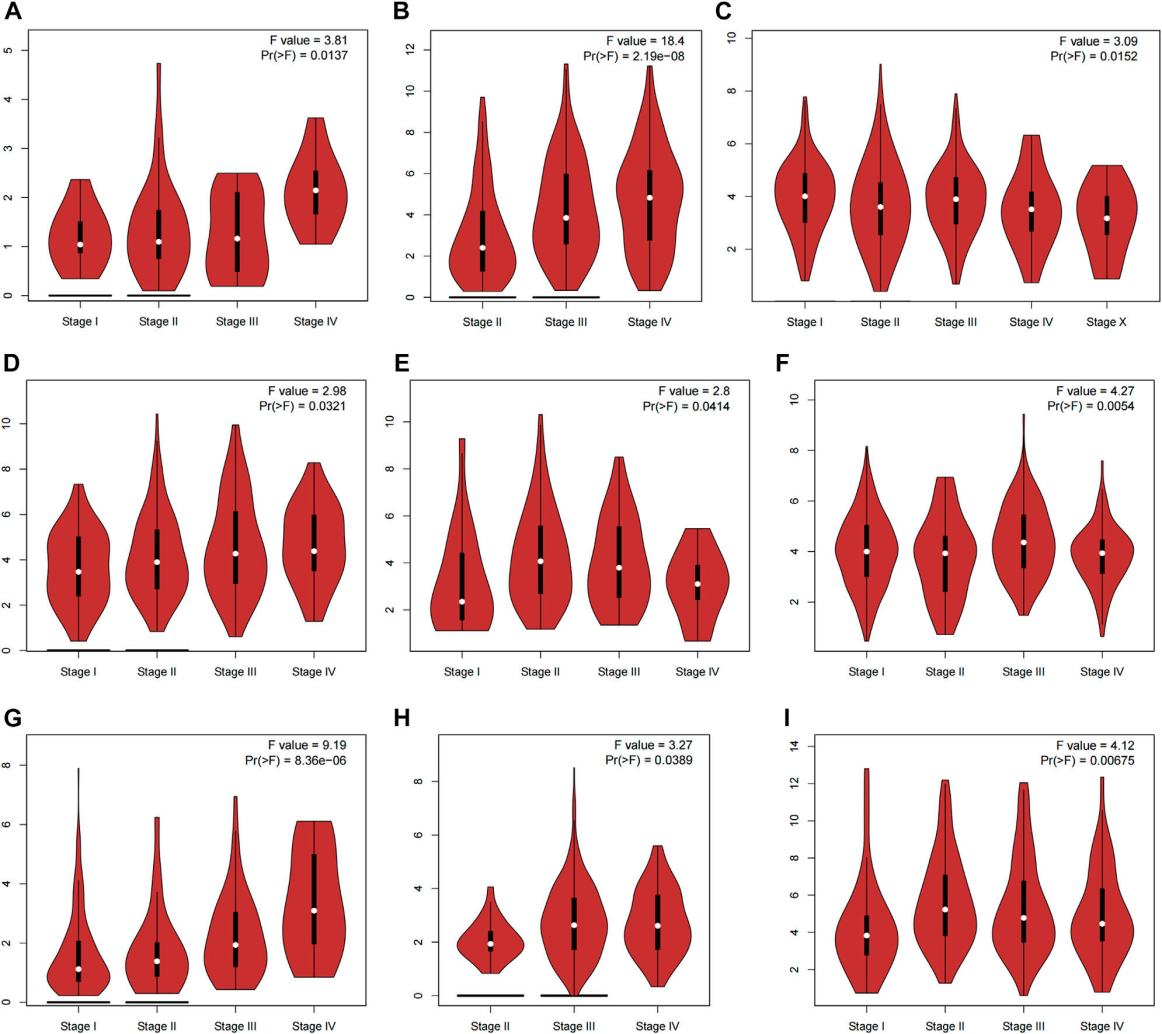
Analysis of CNN1 expression in different tumor stages based on the GEPIA database. **(A)** ACC, **(B)** BLCA, **(C)** BRCA, **(D)** COAD, **(E)** ESCA, **(F)** KIRC, **(G)** KIRP, **(H)** OV, and **(I)** STAD.

We first extracted and analyzed the expression of CNN1 in pan-cancerous tissues from the TIMER, CPTAC, and GEPIA databases. The expression of CNN1 in bladder carcinoma (BLCA), BRCA, cervical squamous cell carcinoma (CESC), COAD, esophageal adenocarcinoma (ESCA), KICH, kidney renal papillary cell carcinoma (KIRP), LUAD, lung squamous cell carcinoma (LUSC), rectum adenocarcinoma (READ), PRAD, STAD, thyroid carcinoma (THCA), and UCEC tumor tissues was significantly lower than that in para-carcinoma tissues, while the protein expression level of CNN1 in CHOL and LIHC tumor tissues was higher than that in para-carcinoma tissues (Figure 1A). Figure 1B shows the results of the CPTAC database analysis, which found that the expression levels of CNN1 were significantly lower in BRCA, OV, COAD, KIRC, UCEC, LUAD, HNSC, and HCC tissues (*p* < 0.01), while in PAAD tissues, the CNN1 expression level was significantly higher than that in normal tissues that were adjacent to the cancer tissues (*p* < 0.01). In addition, to further evaluate the expression of CNN1 in different tumor stages, the CNN1 expression levels were closely associated with the clinicopathological stages of patients with ACC, BLCA, BRCA, COAD, ESCA, KIRC, KIRP, OV, and STAD using the GEPIA database analysis (*p* < 0.05, Figure 2).

### 3.2 Correlation between CNN1 protein expression levels and survival prognosis of cancer patients

**FIGURE 3:**
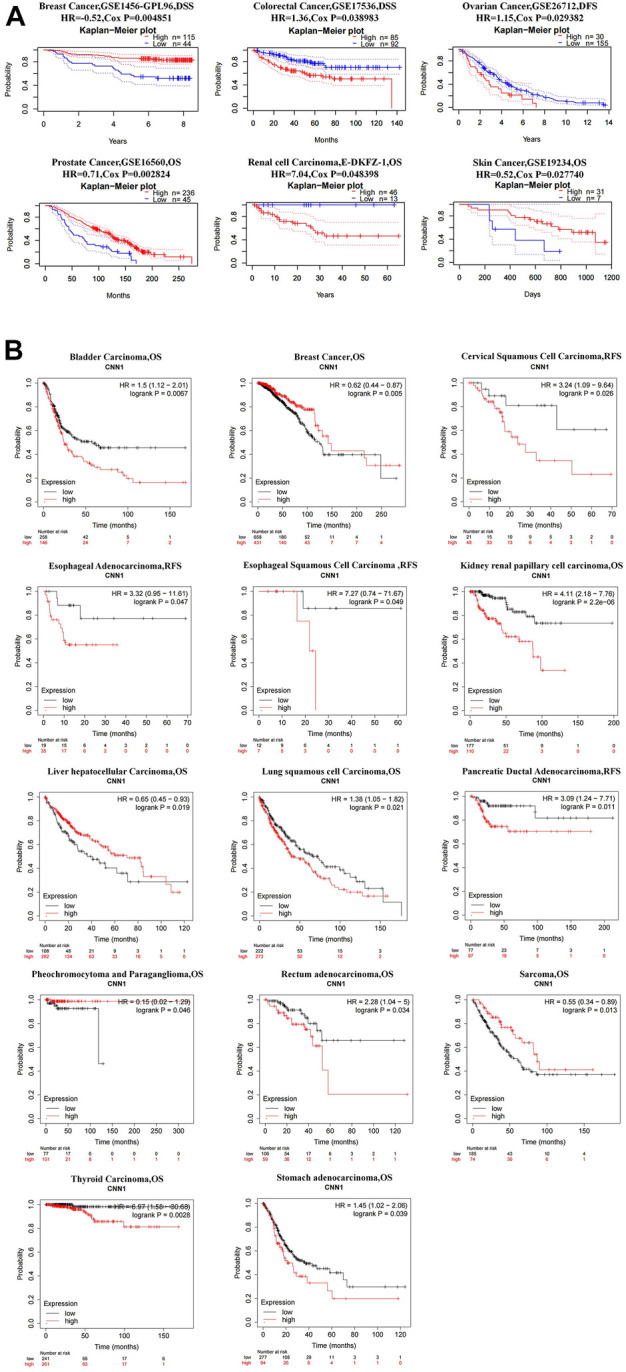
Prognostic analysis of CNN1 in pan-cancer. **(A)** Prognostic analysis of CNN1 in pan-cancer based on the PrognoScan database. **(B)** Prognostic analysis of CNN1 in pan-cancer based on the Kaplan–Meier plotter database.

To analyze the effect of high *versus* low expression of CNN1 on tumor prognosis, we performed a pan-cancer survival analysis using the PrognoScan, Kaplan–Meier plotter, and GEPIA databases. In general, low CNN1 expression had a good prognosis in most of the tumors; high CNN1 expression also had a good prognosis in a small number of tumors. As shown in Figure 2, patients in the CNN1 low-expression group in BLCA, LUSC, READ, THCA, and STAD had better OS than patients in the CNN1 high-expression group (*p* < 0.05). In the case of CESC, ESCA, ESCC, and PAAD, patients in the CNN1 low-expression group had better RFS than those in the CNN1 high-expression group (*p* < 0.05). Moreover, the RFS and OS of patients in the CNN1 low-expression group in KIRP were better than those of patients in the CNN1 high-expression group (*p* < 0.05). It is noteworthy that in BRCA, pheochromocytoma and paraganglioma (PCPG), and SARC, patients in the CNN1 high-expression group had better OS than those in the CNN1 low-expression group (*p* < 0.05). Patients in the CNN1 high-expression group of uterine corpus endometrial carcinoma had better RFS than those in the CNN1 low-expression group (*p* < 0.05, Figure 3).

### 3.4 CNN1 expression correlates with tumor immune and molecular subtypes

**FIGURE 4:**
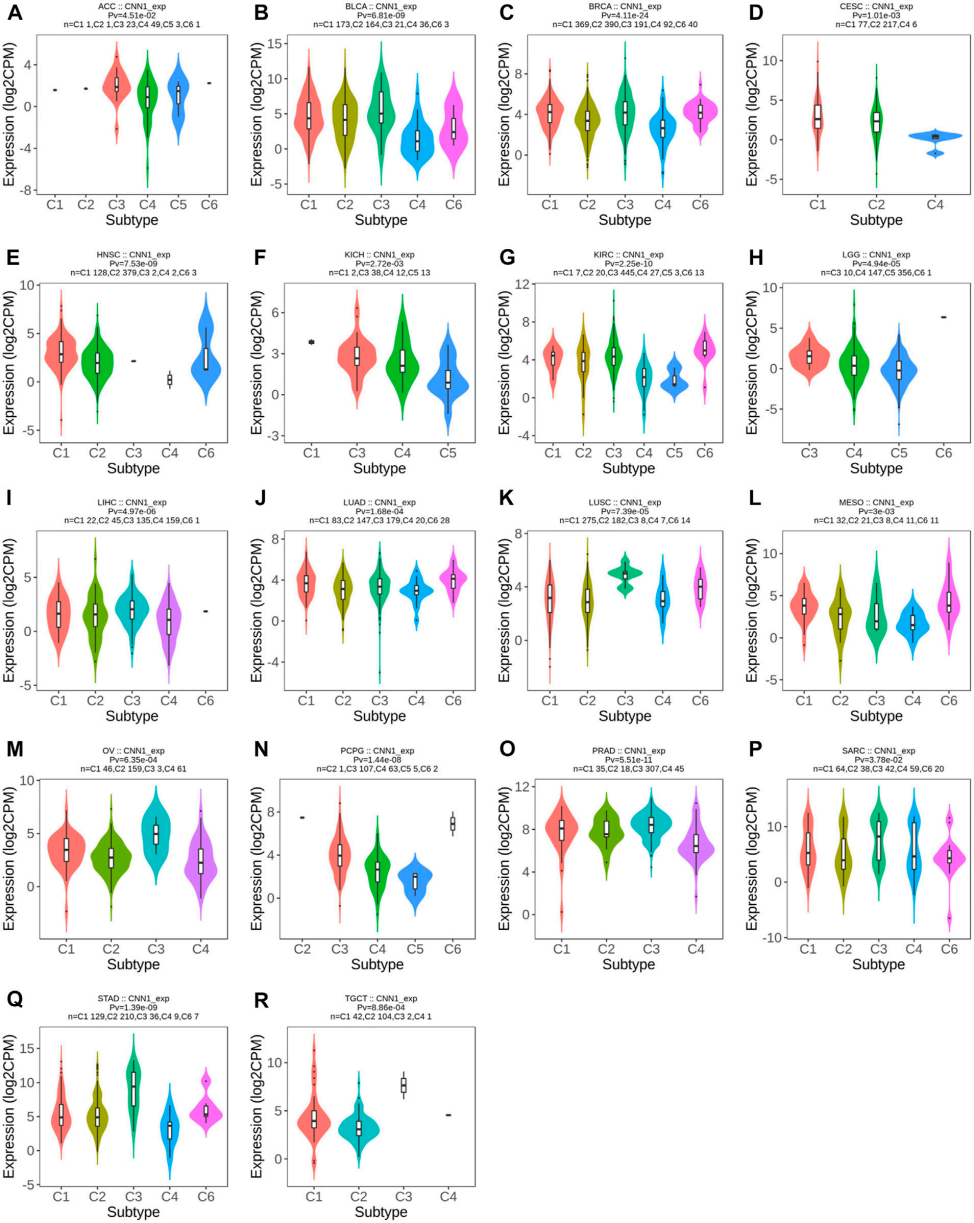
Relationship between CNN1 expression and immune subtypes. **(A)** ACC, **(B)** BLCA, **(C)** BRCA, **(D)** CESC, **(E)** HNSC, **(F)** KICH, **(G)** KIRC, **(H)** LGG, **(I)** LIHC, **(J)** LUAD, **(K)** LUSC, **(L)** MESO, **(M)** OV, **(N)** PCPG, **(O)** PRAD, **(P)** SARC, **(Q)** STAD, and **(R)** TGCT.

**FIGURE 5:**
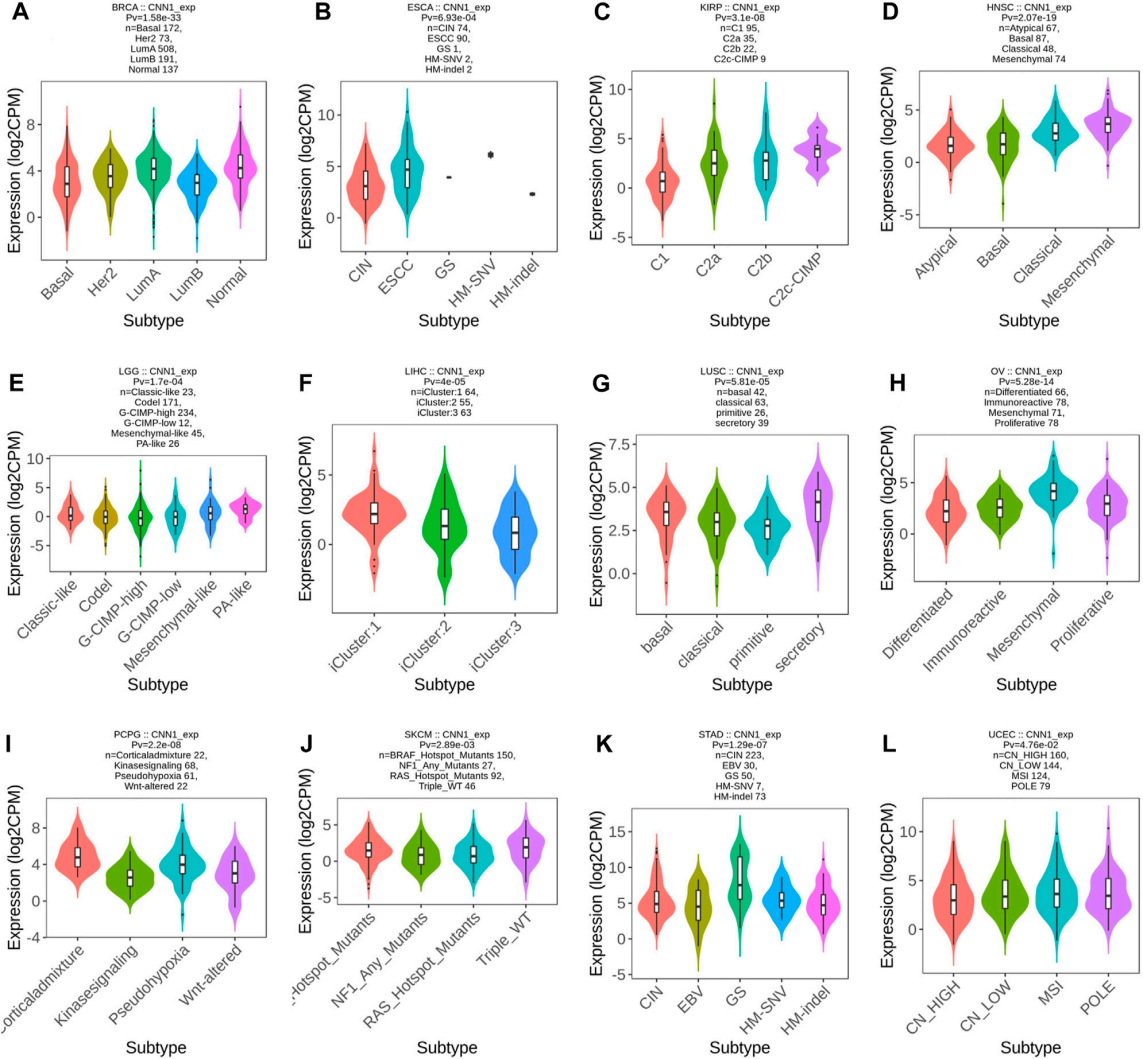
Relationship between CNN1 expression and molecular subtypes. **(A)** BRCA, **(B)** ESCA, **(C)** KIRP, **(D)** HNSC, **(E)** LGG, **(F)** LIHC, **(G)** LUSC, **(H)** OV, **(I)** PCPG, **(J)** SKCM, **(K)** STAD, and **(L)** UCEC.

To explore the potential immunotherapeutic effects of CNN1, we first utilized the TISIDB website to validate the association between CNN1 expression and the immune and molecular subtypes in 37 tumors. The results showed that the CNN1 expression correlated with the immune subtypes of ACC, BLCA, BRCA, CESC, HNSC, KICH, KIRC, LGG, LIHC, LUAD, LUSC, MESO, OV, PCPG, PRAD, SARC, STAD, and TGCT (*p* < 0.05), as detailed in Figures 4A–R. In addition, the expression of CNN1 correlated with the tumor molecular subtypes of BRCA, ESCA, KIRP, HNSC, LGG, LIHC, LUSC, OV, PCPG, SKCM, STAD, and UCEC (*p* < 0.05), as shown in Figure 5. The aforementioned results revealed that the CNN1 expression was associated with both tumor immune and molecular subtypes.

### 3.5 Correlation of CNN1 protein expression levels with immune checkpoint gene expression levels and immune cell infiltration

**FIGURE 6:**
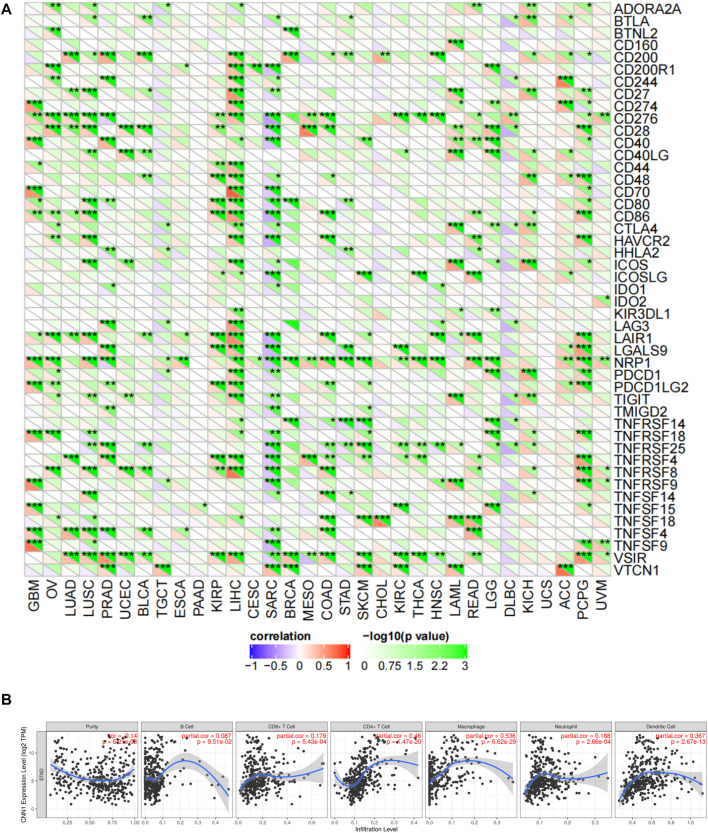
Correlation of CNN1 expression levels with immune checkpoint gene expression levels and immune cell infiltration. **(A)** Correlation of CNN1 expression with 47 immune checkpoint genes in pan-cancer using the Sangerbox online platform. **(B)** Correlation of CNN1 expression with immune cell infiltration in STAD using the TIMER database.

Next, we evaluated the relationship between the expression of CNN1 and 47 immune checkpoint genes through the Sangerbox online platform and found that the expression of CNN1 correlated with GBM, OV, LUAD, LUSC, PRAD, BLCA, KIRP, LIHC, BRCA, COAD, SKCM, KIRC, THCA, HNSC, LAML, READ, LGG, KICH, ACC, PCPG, UVM, SARC, STAD, and DLBC; 47 immune checkpoint genes in tumors were strongly correlated. Among these checkpoint genes, the expression of CNN1 correlated positively with immune checkpoint genes in GBM, OV, LUAD, LUSC, PRAD, BLCA, KIRP, LIHC, BRCA, COAD, SKCM, KIRC, THCA, HNSC, LAML, READ, LGG, KICH, ACC, PCPG, and UVM. This positive correlation was particularly prominent in LIHC and PCGC, where 30 and 23 immune checkpoint genes were positively correlated with the expression of CNN1, respectively. Notably, the expression of CNN1 in SARC and DLBC was negatively correlated with the immune checkpoint genes. This suggests that high CNN1 expression, in most cases, indicates a better prognosis for immunotherapy, but in the immunotherapy of SARC and DLBC, CNN1 inhibitors might achieve a better prognosis (Figure 6A).

Based on this, we used the TIMER database to link CNN1 expression to the immune cell infiltration of STAD. The analysis displayed that the expression of CNN1 was significantly correlated with CD4^+^ T and CD8^+^ T and dendritic cells, which include macrophages and neutrophils in STAD (*p* < 0.01) (Figure 6B).

### 3.6 GSEA enrichment analysis of CNN1

**FIGURE 7:**
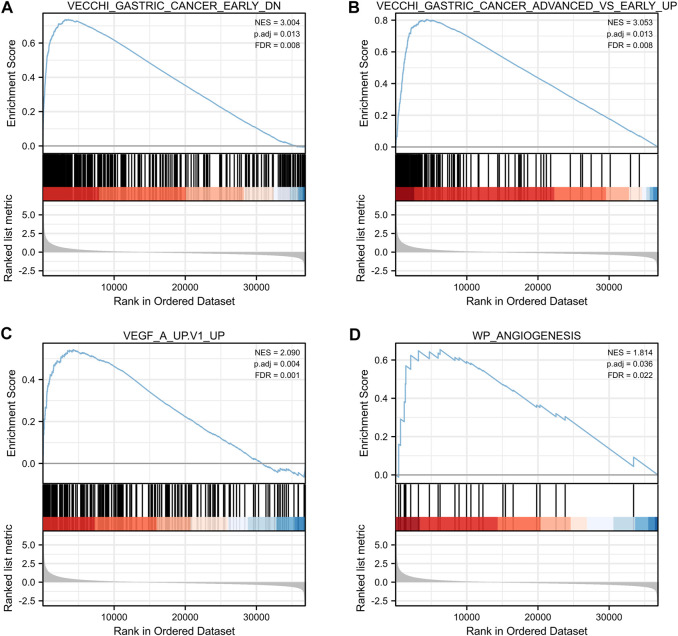
Single gene enrichment analysis of CNN1. **(A)** VECCHI_GASTRIC_CANCER_EARLY_DN. **(B)** VECCHI_GASTRIC_CANCER_ADVANCED_VS_EARLY_UP. **(C)** VEGF_A_UP.V1_UP. **(D)** WP_ANGIOGENESIS.

To further investigate the functional enrichment of CNN1 in GC, we used the ggplot2 package (version 3.3.3) to perform a single gene differential analysis and visualize the results for the following four gene sets: VECCHI_GASTRIC_CANCER_EARLY_DN, VECCHI_GASTRIC_CANCER_ADVANCED_VS_EARLY_UP, WP_ANGIOGENESIS, and VEGF_A_UP.V1_UP, which are the normal tissue and early GC expression downregulated gene set, expression upregulated gene set in early GC *versus* advanced gastric cancer, angiogenesis-related gene set, and upregulated gene set in HUVEC cells (endothelium) treated with VEGFA, respectively. The outcomes revealed positively correlated CNN1 with all of the aforementioned gene sets: VECCHI_GASTRIC_CANCER_EARLY_DN (NES = 3.004; p. adjust = 0.013; FDR = 0.008) (Figure 7A), VECCHI_GASTRIC_CANCER_ADVANCED_VS_EARLY_UP (NES = 3.053; p. adjust = 0.013; FDR = 0.008) (Figure 7B), VEGF_A_UP.V1_UP (NES = 2.090; p. adjust = 0.004; FDR = 0.001) (Figure 7C), and WP_ANGIOGENESIS (NES = 1.814; p. adjust = 0.036; FDR = 0.022) (Figure 7D).

### 3.7 HE staining and immunohistochemical study of CNN1 in tumor and paraneoplastic tissues

**FIGURE 8:**
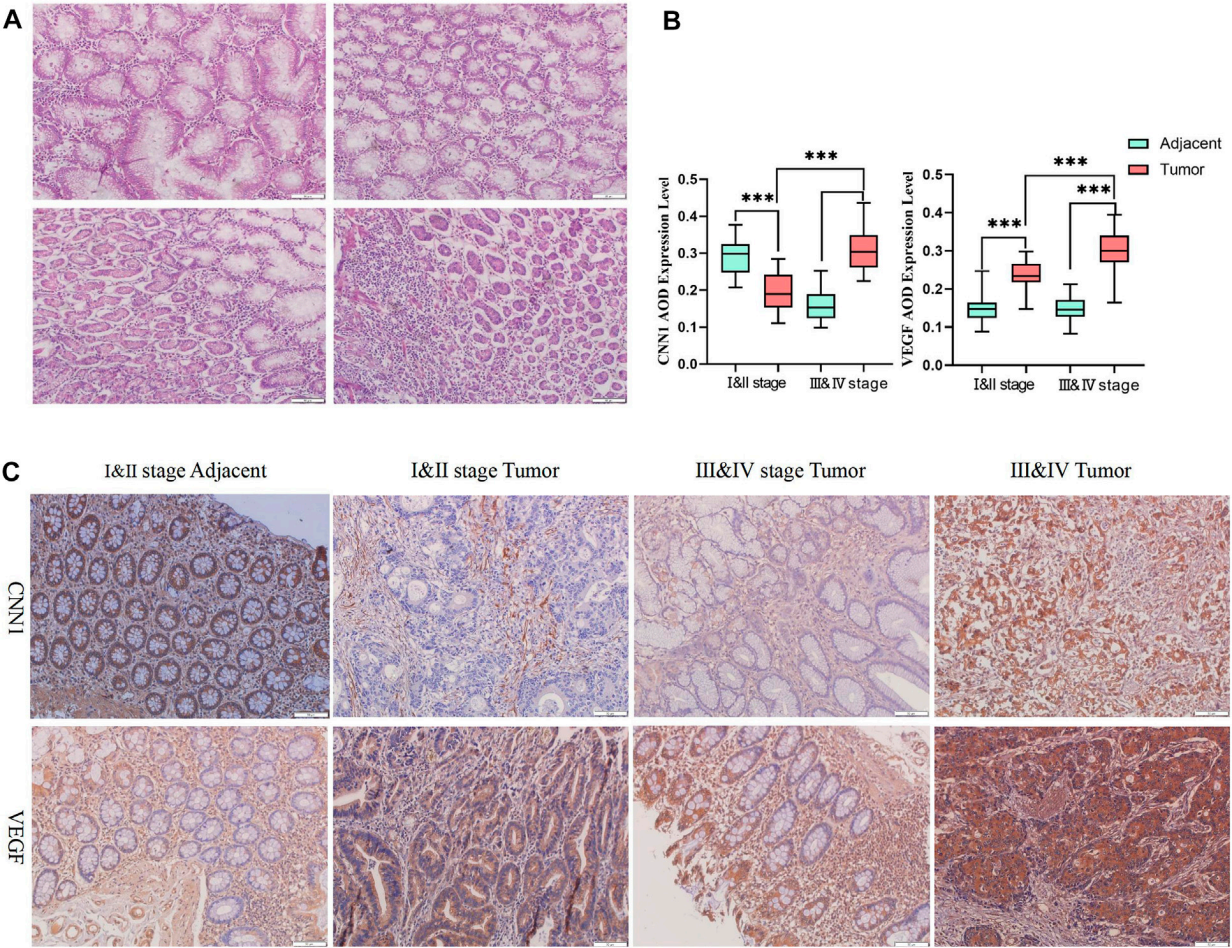
HE staining and immunohistochemistry results. **(A)** HE staining results in tumor and paraneoplastic tissues in GC. **(B)** Statistical results of immunohistochemical experiments (*p < 0.05, **p < 0.01, and ***p < 0.001). **(C)** Immunohistochemical results of CNN1 and VEGF between tumor and paraneoplastic tissues in GC.

The HE staining results showed that the paraneoplastic tissues were lined with the nuclei. The nuclei were small, round, and located at the base. The cells were full and uniform in size, and a large number of cup-shaped cells were observed. The tumor tissue was filled with tumor cells in the submucosal lymphatic vessels, and the adenoma cells were extending and growing from the surface to the normal cells; inflammatory infiltration was present. The tumor cells were morphologically diverse, with concentrated deep-stained and crowded nuclei, severely disturbed nuclear arrangement, and an obvious degree of heterogeneity (Figure 8A). According to the immunohistochemical results, CNN1 expression in paraneoplastic tissues was significantly higher than in tissues from early tumors. When comparing unpaired samples, we found that CNN1 expression in early GC tissues was significantly lower than its expression in advanced GC tissues. Furthermore, the expression of VEGF in paraneoplastic tissues was significantly lower than that in tumor tissues, while when comparing with unpaired samples of early GC tissues and advanced GC tissues, VEGF was significantly highly expressed in advanced GC samples (Figures 8B, C).

### 3.8 Expression of CNN1 correlates with the clinical prognosis of GC

**FIGURE 9:**
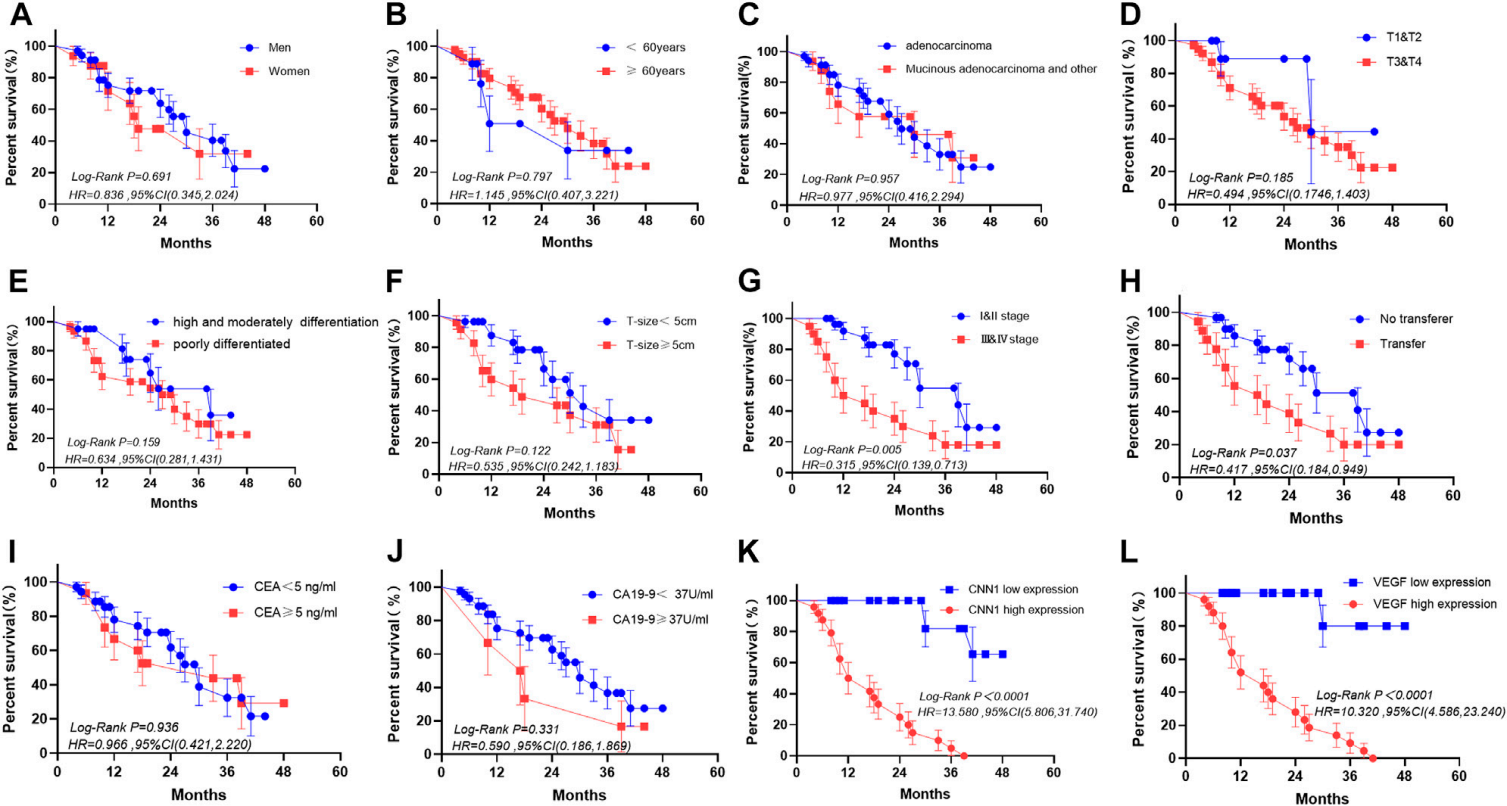
Prognostic analysis of clinicopathologic characteristics in gastric cancer. **(A)** Sex. **(B)** Age (year). **(C)** Histological type. **(D)** Depth of tumor invasion. **(E)** Degree of differentiation. **(F)** Tumor size. **(G)** TNM staging. **(H)** Lymph node metastasis. **(I)** CEA. **(J)** CA 19-9. **(K)** Expression of CNN1. **(L)** Expression of VEGF.

Based on the median values of CNN1 and VEGF expressions, we separated patients into high- and low-expression groups and evaluated the correlation between CNN1 and VEGF expressions in tumor and para-cancer tissues from clinical gastric cancer patients and also various clinical characteristics. According to the results, there were no significant differences between the two groups in terms of age, sex, histological type, differentiation, tumor size, and CEA and CA 19-9 levels, except for the depth of tumor invasion, TNM stage, and lymph node metastasis. (Tables 1, 2). In the subsequent Kaplan–Meier survival analysis, we found that high expression of CNN1 was associated with a poorer survival prognosis (*p* < 0.001, HR = 13.580, 95% CI [5.806–31.740]), and high expression of VEGF was also associated with a poorer survival prognosis (*p* < 0.001, HR = 10.320, 95% CI: [4.586–23.240], Figure 9). We performed a univariate analysis of the clinicopathological factors affecting prognosis using Cox regression models, which showed that the TNM staging (*p* = 0.009, HR = 2.867, 95% CI [1.299–6.324]), lymph node metastasis (*p* = 0.046, HR = 2.198, 95% CI [1.015–4.758]), a CNN1 expression level (*p* < 0.001, HR = 23.541, 95% CI [5.438–101.901]), and the VEGF expression level (*p* < 0.001, HR = 19.688, 95% CI [4.607–84.131]) were associated with a poorer prognosis (Table 3).

**TABLE 1:** Correlation between CNN1 and clinicopathologic characteristics.

Clinicopathologic characteristics		n	CNN1 expression		*p-*value
			Low	High	
Sex	Male	34	19	15	0.423
	Female	16	7	9	
Age (years)	<60	9	5	4	0.814
	≥60	41	21	20	
Histological type	Adenocarcinoma	34	17	17	0.680
	Mucoid adenocarcinoma	16	9	7	
Depth of tumor invasion	T1 and T2	12	10	2	0.013
	T3 and T4	38	16	22	
Degree of differentiation	High and moderately differentiated	20	12	8	0.355
	Poorly differentiated	30	14	16	
Tumor size	<5 cm	27	16	11	0.266
	≥5 cm	23	10	13	
TNM staging	Ⅰ and Ⅱ stage	30	28	8	<0.001
	Ⅲ and Ⅳ stage	20	4	16	
Lymph node metastasis	Without	32	22	10	0.002
	With	18	4	14	
CEA	<5 ng/ml	35	20	15	0.266
	≥5 ng/ml	15	6	9	
CA 19-9	<37 U/ml	44	29	19	0.065
	≥37 U/ml	6	1	5	

**TABLE 2:** Correlation between VEGF and clinicopathologic characteristics.

Clinicopathologic characteristics		n	VEGF expression		*p-*value
			Low	High	
Sex	Male	34	18	16	0.544
	Female	16	7	9	
Age (years)	<60	9	5	4	0.713
	≥60	41	20	21	
Histological type	Adenocarcinoma	34	16	18	0.544
	Mucoid adenocarcinoma	16	9	7	
Depth of tumor invasion	T1 and T2	12	10	2	0.008
	T3 and T4	38	15	23	
Degree of differentiation	High and moderately differentiated	20	12	8	0.248
	Poorly differentiated	30	13	17	
Tumor size	<5 cm	27	16	11	0.156
	≥5 cm	23	9	14	
TNM staging	Ⅰ and Ⅱ stage	30	21	9	0.001
	Ⅲ and Ⅳ stage	20	4	16	
Lymph node metastasis	Without	32	21	11	0.003
	With	18	4	14	
CEA	<5 ng/ml	35	19	16	0.355
	≥5 ng/ml	15	6	9	
CA 19-9	<37 U/ml	44	24	20	0.082
	≥37 U/ml	6	1	5	

**TABLE 3:** Single-factor Cox regression analysis.

Clinicopathologic characteristics		Hazard ratio	95.0% CI for exp. (B)		*p*-value
			Lower	Upper	
Sex	Male	1	0.51	2.742	0.696
	Female	1.182			
Age (years)	<60	1	0.332	2.345	0.882
	≥60	0.801			
Histological type	Adenocarcinoma	1	0.443	2.361	0.958
	Mucoid adenocarcinoma	1.023			
Depth of tumor invasion	T1 and T2	1	0.595	10.803	0.208
	T3 and T4	2.536			
Degree of differentiation	High and moderately differentiated	1	0.673	3.843	0.29
	Poorly differentiated	1.608			
Tumor size	<5 cm	1	0.832	3.984	0.13
	≥5 cm	1.82			
TNM staging	Ⅰ and Ⅱ stages	1	1.299	6.324	0.009
	Ⅲ and Ⅳ stages	2.867			
Lymph node metastasis	Without	1	1.015	4.758	0.046
	With	2.198			
CEA	<5 ng/mL	1	0.458	2.333	0.937
	≥5 ng/mL	1.034			
CA 19-9	<37 U/mL	1	0.581	4.142	0.381
	≥37 U/mL	1.551			
CNN1	Low expression	1	5.438	101.901	0.000
	High expression	23.541			
VEGF	Low expression	1	4.607	84.131	0.000
	High expression	19.688			

## 4 Discussion

CNN1 plays an important role in the differentiation and contraction of smooth muscle cells, and it is also a marker of smooth muscle cells during differentiation and maturation. CNN1 is mainly located in the cytoskeleton and focal adhesion. In most tumors, CNN1 promotes further malignancy. In bladder cancer, it promotes tumor development and metabolic reprogramming through the HIF-1α pathway (Zhang et al., 2022). Also, it was recently found that high CNN1 expression mediated the development of chemoresistance by inducing cancer-associated fibroblasts-mediated matrix stiffness in gastric cancer (Lu et al., 2023). However, by suppressing CNN1, metastasis in breast cancer is promoted (Wang et al., 2020). CNN1 inhibits tumor cell proliferation and migration probably by stabilizing the cytoskeleton and making intercellular junctions tighter. However, a high level of CNN1 promotes anoxia and angiogenesis in the tumor microenvironment, which increase the degree of tumor malignancy. CNN1 serves as a pivotal regulator for smooth muscle contraction under the physiological condition, while it is presented as a double-edged sword in a complex context under cancerous conditions, which might depend on its expression level and tissue specificity (Miano and Olson, 1996; Samaha et al., 1996; Horiuchi et al., 1999; Yamamura et al., 2007). Nevertheless, the effect of CNN1 involving the initiation and progression of cancers remains largely unknown. In this study, we found that the expression of CNN1 was higher in normal tissue than in pan-cancer tissues, yet this expression would rebound during the progression of cancers. This rebounding expression of CNN1 would regulate angiogenesis and the immune checkpoint to contribute to cancer progression and poor prognosis.

In this pan-cancer research on CNN1, first, we investigated the expression of CNN1 in various tumors by utilizing databases such as TIMER, CPTAC, and GEPIA. Our results showed that the expression of CNN1 was reduced in the pan-cancer tissues, but it was inversed in the CHOL or LIHC. However, previous studies have suggested that CNN1 was found to be elevated in tumor tissues, at least those such as LUSC, OV, LIHC, and BLCA; thus, it might be considered a potential oncogene to be involved in cancer progression (Sasaki et al., 2002; Yamane et al., 2015; Liu et al., 2019; Liu et al., 2021). Furthermore, in using the GEPIA database, we revealed that CNN1 expression levels were closely correlated with the clinicopathological stages of patients with ACC, BLCA, BRCA, COAD, ESCA, KIRC, KIRP, OV, and STAD. It is noteworthy that CNN1 decreased only in BRCA from the early to advanced tumor stages, while in the remaining eight tumor types, the expression of CNN1 in advanced tumor tissues was higher than it was in early tumor tissues, which coincides with the findings of a study correlating MYL9 and CNN1 with the recurrence of colorectal cancer. These findings suggest that CNN1, an oncogene in normal human tissues, is transformed into a pro-oncogenic factor with tumor progression or due to changes in the tumor microenvironment (Qiu et al., 2020). The specific mechanisms involved have to be further investigated, but CNN1 certainly plays a role in the development of most tumors, and this role may be played throughout the stages of tumor development, including through the pre, early, and advanced cancer stages.

In addition, the expression of CNN1 in the advanced stage of pan-cancer tissues was higher than that in the early stage, though it was reversed in BRCA cancer. Furthermore, our results showed that the expression of CNN1 positively correlated with poor prognosis in all 10 types of cancer, including bladder carcinoma. Indeed, a high CNN1 expression was related to poor prognosis in colorectal cancer (Lee et al., 2020; Zhou et al., 2020). On the contrary, the overexpression of CNN1 showed a significant decrease in motility and proliferation of human fibrosarcoma HT1080 cells (Takeoka et al., 2002). CNN1 overexpression also inhibited BRCA carcinogenesis (Wang et al., 2020). These findings are consistent with our results and together suggest that CNN1 is a promising prognostic marker for pan-cancer screening.

Accumulating evidence has highlighted that immunotherapy alone or combined with chemotherapy, radiotherapy, and targeted therapy can largely and significantly improve cancer treatment (Galluzzi et al., 2015; Yu et al., 2019; Padmanabhan et al., 2020). Immune checkpoint blockade, as one of the most promising therapeutic strategies, is also being evaluated in clinical application (Tray et al., 2018). Therefore, we first surveyed the relevance of CNN1 expression to the immune and molecular subtypes of 37 tumors, which included 47 immune checkpoint genes. Our results showed that the expression of CNN1 was correlated with the immune subtypes of 18 tumors (including ACC) and molecular subtypes of 14 tumors (including BRCA). Moreover, CNN1 was strongly correlated with 47 immune checkpoint genes in most tumors. In particular, our results further showed that the expression of CNN1 was significantly correlated with CD4^+^ T cells, CD8^+^ T cells, and dendritic cells and macrophages and neutrophils in STAD (*p* < 0.01). Collectively, our findings indicate a potential and promising immunotherapeutic target for CNN1.

During this research, we noted a significant increase in CNN1 expression in paraneoplastic tissue of gastric adenocarcinoma. Furthermore, in the comparison of tumor stages of gastric adenocarcinoma, CNN1 expression was higher in advanced gastric adenocarcinoma than in early-stage gastric adenocarcinoma. In the prognostic analysis, we also found that in gastric adenocarcinoma, the prognosis of the CNN1 low-expression group was better than that of the high-expression group. This non-linear expression of CNN1 in precancerous tissues or early tumor tissue is particularly evident in gastric adenocarcinoma. In order to understand the molecular mechanism involved in this phenomenon, we performed a single-gene differential analysis of four related gene sets using the R software. We found that CNN1 was indeed positively correlated with normal tissue, early GC expression downregulated gene sets, and the early GC and advanced GC expression upregulated gene sets, which is consistent with our description of its non-linear expression throughout tumor progression. Furthermore, in the angiogenesis-related GSEA analysis, we found that CNN1 was positively correlated with the angiogenesis-related genome and the upregulated gene composition of HUVEC cells (endothelial cells) treated with VEGFA, which might imply that the synergistic effect of CNN1 and VEGFA drives tumor progression. Angiogenesis is an important part of maintaining the tumor microenvironment and creating conditions for tumor cell escape and distal lesion formation (Hanahan and Weinberg, 2011). CNN1, as the earliest actin-related protein isolated from the vascular smooth muscle, is an important factor affecting vascular smooth muscle generation (Takahashi et al., 1988; Matthew et al., 2000; Takahashi and Yamamura, 2003; Babu et al., 2006). Both positive and negative expressions of CNN1 in tumor vascular smooth muscle have been reported (Verone et al., 2013), where CNN1 expression is reduced in the early stages of hepatocellular carcinoma, which is in agreement with the observations in this research. The results suggest that CNN1 has a crucial role in angiogenesis and maturation, and CNN1 expression is correlated with the stability of the tumor cytoskeleton (Emens, 2012), which also influences the migration of tumor cells and the formation of distal lesions together with VEGFA. We speculate that the synergistic effect of CNN1 and VEGF accelerates the formation of the tumor microenvironment, which in turn influences their expressions. To further enhance the credibility of the GESA results, we included 50 clinical GC samples in our immunohistochemical analyses to detect the expression of CNN1 and VEGF in clinical GC tissues. The experimental results are also compatible with the abovementioned findings. Moreover, in the analysis of the clinical data of these 50 patients, we found that TNM staging, lymph node metastasis, and CNN1 and VEGF expressions were all prognostic risk factors, which also indicates that the high expressions of CNN1 and VEGF may promote the metastasis and progression of tumor cells. Therefore, CNN1 and VEGF are potential prognostic risk factors for survival.

Based on the TIMER database, we further investigated the relevance of CNN1 to immune cell infiltration in STAD. The results showed that CNN1 expression was significantly correlated with CD4^+^ T, CD8^+^ T, and dendritic cells and macrophages and neutrophils in STAD (*p* < 0.01), which not only allowed us to see CNN1 as a relevant prognostic factor in a variety of tumors, such as GC, but also showcases the promise of CNN1 as a target for immunotherapy.

In conclusion, our results show that CNN1 plays multiple roles in tumor angiogenesis and the immune checkpoint by contributing to the progression of and poor prognosis in various cancers, thus providing a promising candidate for pan-cancer prognosis and immunotherapy. Our results also suggest that CNN1 serves as a double-edged sword between the physiological and pathological conditions, or between cancer initiation and progression, and it might also vary on the type of cancer, which might provide precautions and evidence for CNN1-targeted drug development and its clinical application.

## 5 Limitations

Although we made some exploration of the prognostic role, immunotherapeutic potential, and possible mechanisms of action of CNN1 from a pan-cancer perspective using multiple databases and multiple analytical tools, there were still some limitations. First, due to the information collected from the various databases, the present study had some systematic biases, and we aim to overcome this in future studies by collecting clinical tumor samples and performing single-cell sequencing to obtain higher-resolution data. Second, although *in vitro* studies clearly show that phosphorylation regulates the function of CNN1 (Liu and Jin, 2016), this study did not address the effect of phosphorylation on CNN1 function, and perhaps the alteration of CNN1 function during tumor development may be investigated by looking at the relationship between the phosphorylation status and degradation of CNN1. Third, this study was focused on CNN1 bioinformatics analysis and clinical sample detection, but it was slightly weak in strength, and we may add *in vitro* cellular experiments or *in vivo* animal experiments to investigate the molecular mechanisms of CNN1 in cancer development in more detail. Fourth, although we have confirmed the great potential of CNN1 in pan-cancer immunotherapy, both inhibition and overexpression of CNN1 were found to be relevant in different tumors; therefore, more research has to be done to implement the use or targeting of CNN1 in tumor immunotherapy.

## Data Availability

The original contributions presented in the study are included in the article/Supplementary Material; further inquiries can be directed to the corresponding authors.
